# Superior labrum anterior to posterior lesions: Part 1 – Imaging and anatomy with arthroscopic classification

**DOI:** 10.4102/sajr.v27i1.2706

**Published:** 2023-10-26

**Authors:** Peter Mercouris, Matthew Mercouris

**Affiliations:** 1 Diagnostic Radiologist, Lake, Smit & Partners, Gateway Private Hospital, Durban, South Africa; 2 Department of Orthopaedics, Mitchell’s Plain District Hospital, Cape Town, South Africa

**Keywords:** shoulder, glenoid labrum, SLAP lesions or tears, MRI arthrography, anatomic variants of the labrum

## Abstract

The glenoid labrum deepens the glenoid fossa and allows for the attachment of the long head of the biceps tendon and the glenohumeral ligaments, contributing to the stability of the glenohumeral joint. The superior labrum is a common site of labral injury, especially in athletic activities involving overhead activity. The acronym SLAP (superior labrum anterior to posterior or anteroposterior) lesion was introduced by Snyder and colleagues in 1990 to describe superior labral tears. The article will review the optimal technique to image the glenoid labrum, covering normal labral anatomy with special emphasis on the anatomic variants of the labrum that can be mistaken for SLAP tears by means of colour illustrations, magnetic resonance imaging and correlative arthroscopy images.

**Contribution:**

The illustrated review functions as a crucial radiological guide for both radiologists and orthopaedic surgeons. The combination of illustrations, MR and correlative arthroscopic images enhances the comprehension of normal labral anatomy and its variants. The review underscores the significance of understanding anatomic variations that may be misinterpreted as pathology. This understanding is vital in guiding orthopaedic management for patients, ensuring appropriate treatment strategies.

## Introduction

**FIGURE 1:**
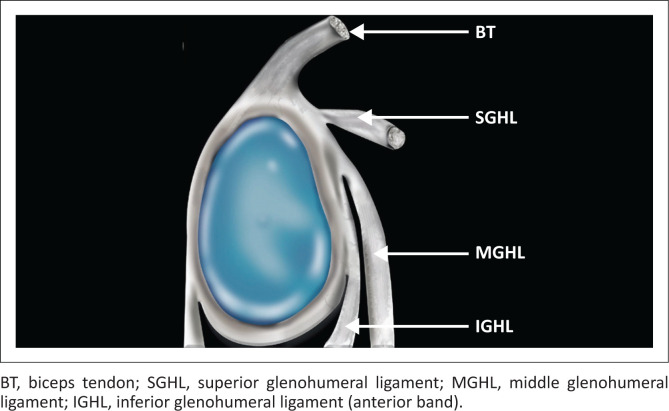
Colour illustration of the glenoid labroligamentous anatomy.

The glenoid labrum is a cuff of fibrocartilagenous tissue that surrounds the glenoid cavity. It functions to deepen the glenoid fossa and allows for the attachment of the long head of the biceps brachii tendon and glenohumeral ligaments (Figure 1), contributing to stability of the glenohumeral joint.^1^ Tears of the superior labrum are a common cause of labral pathology. The acronym SLAP, which denotes the superior labrum from anterior to posterior relative to the biceps anchor, was introduced in 1990 by Snyder et al.^2^ to describe lesions of the superior labrum of the shoulder based on arthroscopic evaluation. A superior labrum anterior to posterior or anteroposterior (SLAP) lesion originates at the site of attachment of the long head of the biceps tendon but can extend to the anterior or posterior portion of the labrum and regional structures. Snyder’s classification described four types of SLAP lesions, but with the descriptions of additional SLAP lesions by several authors, the original classification has been expanded and there are currently 10 types of SLAP lesions.^3^

The arthroscopic prevalence of SLAP lesions in original studies ranges from 3.9% to 11.8%,^2,4^ but a more recent study has shown the prevalence to be as high as 26%.^5^ Arthroscopy remains the gold standard for the diagnosis of a SLAP lesion. The clinical diagnosis of a SLAP lesion is difficult, and patients often present with non-specific shoulder pain particularly with overhead or cross-body motion.^6^ MRI has been shown to be accurate in diagnosing SLAP lesions and therefore plays an important role in diagnosis.

This article will review the optimal imaging technique and pictorially illustrate the labral anatomy and the anatomic variants of the labrum by means of colour illustrations, MRI images and correlative arthroscopy images. Knowledge of this anatomy is necessary to accurately assess the labrum for a SLAP lesion.

## Imaging techniques

The glenoid labrum can be imaged by MRI, MRI arthrography (direct and indirect) and CT arthrography. Ultrasound cannot reliably image the superior labrum. Direct MRI arthrography is the most reliable and accurate modality in the assessment of superior labral pathology with a sensitivity of 82% – 100%, a specificity of 69% – 98% and an accuracy of 74% – 94%.^6,7^ Conventional or unenhanced MRI demonstrates a sensitivity, specificity and accuracy of 66% – 98%, 71% – 90% and 77% – 96%, respectively, whereas indirect MRI arthrography demonstrates a sensitivity, specificity and accuracy of 84% – 91%, 58% – 85% and 78% – 89%, respectively.^7^ CT arthrography has a sensitivity of 94% – 97%, a specificity of 73% – 77% and an accuracy of 86%.^7^

A meta-analysis study by Symanski et al.^8^ confirmed the superiority of direct MR arthrography over conventional MRI in the diagnosis of SLAP tears. The study also concluded that overall, 3-Tesla (3T) imaging appears to improve diagnostic accuracy for SLAP tears compared with 1.5T MRI. An additional conclusion was that 3T imaging, both with direct MR arthrography and conventional MRI, appears to have a higher diagnostic accuracy compared to 1.5T imaging.^8^

Direct MRI arthrography is superior to conventional MRI in that it involves the intra-articular administration of gadolinium-based contrast which results in capsular distension and improved visualisation of the capsulolabral structures, including tears. The gadolinium-based contrast can be introduced into the glenohumeral joint under fluoroscopic or ultrasound guidance. The disadvantage of this technique is its relative invasive nature with the small risk of infection, bleeding and post-procedural pain.^9^ Indirect MRI arthrography involves the intravenous administration of gadolinium-based contrast, and this may be supplemented with post-injection shoulder exercise. The advantage of this method is its non-invasive nature, but the major disadvantage is the absence of capsular distension as well as enhancement of all the intra-articular vascularised structures, with the latter possibly resulting in overestimation of pathology.^9,10^ CT arthrography is, in the author’s institution, reserved for patients in whom MRI is contraindicated. The major disadvantages include the use of ionising radiation and reduced accuracy in the assessment of the glenohumeral ligaments, rotator cuff, paralabral cysts and bone marrow.^7^

A typical direct MR arthrogram protocol would include fat-saturated T1-weighted sequences in three planes (coronal oblique, sagittal oblique and axial) supplemented with a fat-saturated T2 weighted coronal oblique sequence and non-fat-saturated T1-weighted sagittal sequence in the neutral position. Additional imaging, if tolerated by the patient, with the arm placed in the abduction and external rotation (ABER) position is useful in detecting subtle anteroinferior labral tears and tears of the undersurface of the rotator cuff.^11^ This sequence is obtained as a fat-saturated T1-weighted sequence. A typical conventional (non-arthrogram) MRI protocol would include fat-saturated proton density weighted sequences in three planes (coronal oblique, sagittal oblique and axial), coronal oblique fat-saturated T2-weighted sequence and a sagittal oblique non-fat saturated T1-weighted sequence.

## Labral anatomy

**FIGURE 2:**
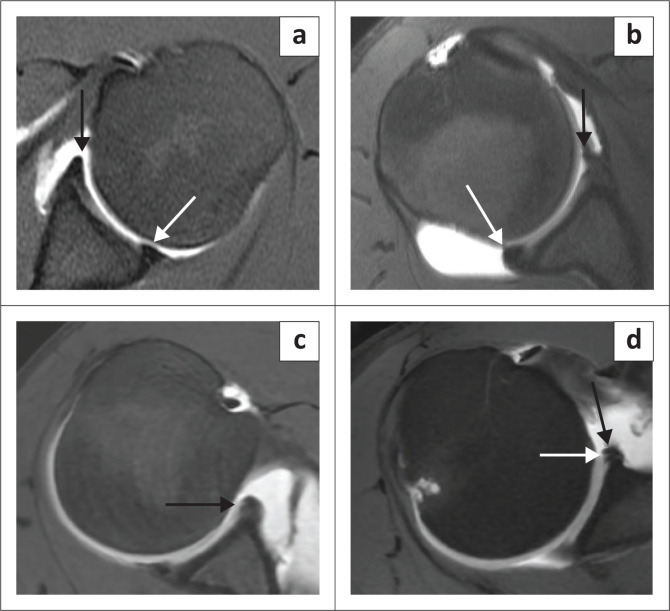
Labral morphology – shape: (a) Triangular anterior labrum (black arrow) and blunted posterior labrum (white arrow). (b) Crescent-like anterior labrum (black arrow) and round posterior labrum (white arrow). (c) Notched anterior labrum (black arrow). (d) Apparent notched appearance to labrum because of close apposition of the inferior glenohumeral ligament (black arrow) to the anterior labrum (white arrow).

There are individual variations of the labrum, particularly related to its size, signal intensity, shape and attachment to the glenoid. The labrum usually measures 3 mm in thickness and 4 mm in width but its size can vary between 2 mm and 14 mm making the criterion of size of no practical diagnostic value.^12,13^ The labrum is usually of low signal intensity on all pulse sequences but increased labral signal can be seen with the magic angle effect, as a variant of the normal, in older individuals, early degeneration or chronic post traumatic change.^14^ The clinical significance of this high signal is thus uncertain especially if the underlying labral morphology is normal.^14^ On transverse sections (axial imaging), the labrum is most commonly triangular in shape^15^ but may be rounded, blunted, cleaved, notched, crescent shaped and even absent (Figure 2).^15,16^ A notched appearance is often attributable to the close apposition of the middle and inferior glenohumeral ligaments (IGHL) to the anterior labrum (Figure 2d).^17^

**FIGURE 3:**
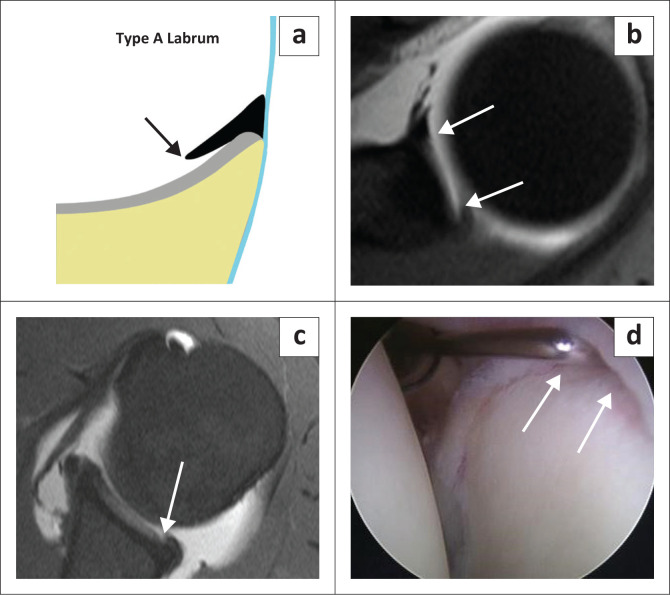
Labral morphology – attachment: Type A labrum: (a) Colour illustration of the central free edge of the labrum. (b) FS T1W MR arthrogram axial view demonstrates the central free edge of the labrum (white arrows). (c) FS T1W MR arthrogram axial view in another patient demonstrating a type A labrum (white arrow) subsequently confirmed arthroscopically (white arrows in d).

**FIGURE 4:**
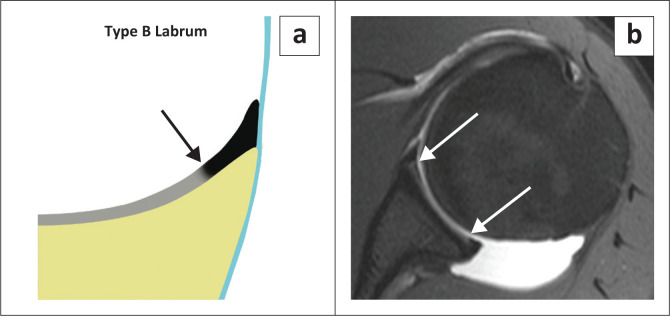
Labral morphology – attachment: Type B labrum: (a) Colour illustration of the firm central attachment of the labrum. (b) FS T1W MR arthrogram axial view demonstrates the firm central (white arrows) attachment of the labrum.

There are two types of labral attachments with the type A labrum (Figure 3) having a central free edge and attached peripherally and the type B labrum (Figure 4) attached both centrally and peripherally.^18^ A type A labral attachment can be mistaken for a labral tear but can be differentiated from a tear in that with the type A attachment, intra-articular contrast (or joint fluid) extends into a smooth tapering recess between the labrum and glenoid cartilage and does not traverse the labrum.

The epiphyseal line represents the junction of the upper and middle thirds of the glenoid fossa body and in general, the labrum is more firmly attached below the epiphyseal line (equator) and has a more variable attachment above this line. The coracoid process tip is a useful landmark to identify the equator.^13^

**FIGURE 5:**
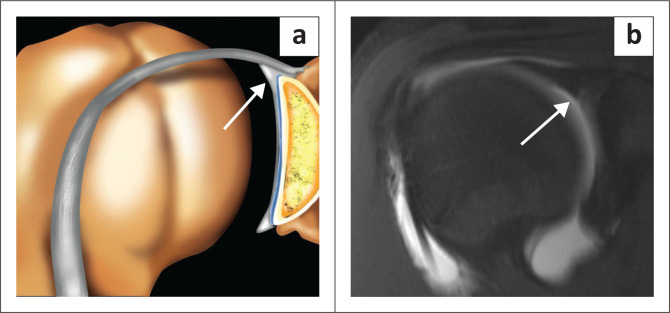
Type 1 biceps labral complex with firm attachment to superior glenoid rim as arrowed in the colour illustration (a) and the FS T1W MR arthrogram coronal oblique view (b).

**FIGURE 6:**
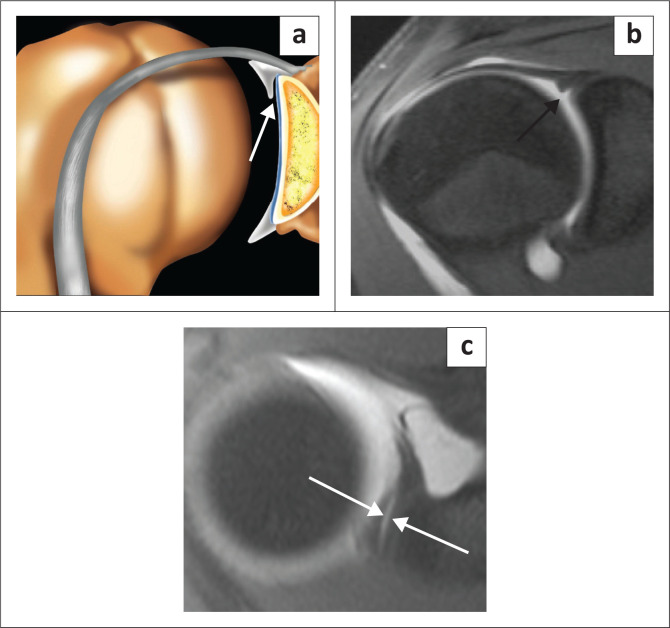
Type 2 biceps labral complex with a small recess between the labrum and cartilage as arrowed in the colour illustration (a), FS T1W MR arthrogram coronal oblique view (b) and axial view (c).

**FIGURE 7:**
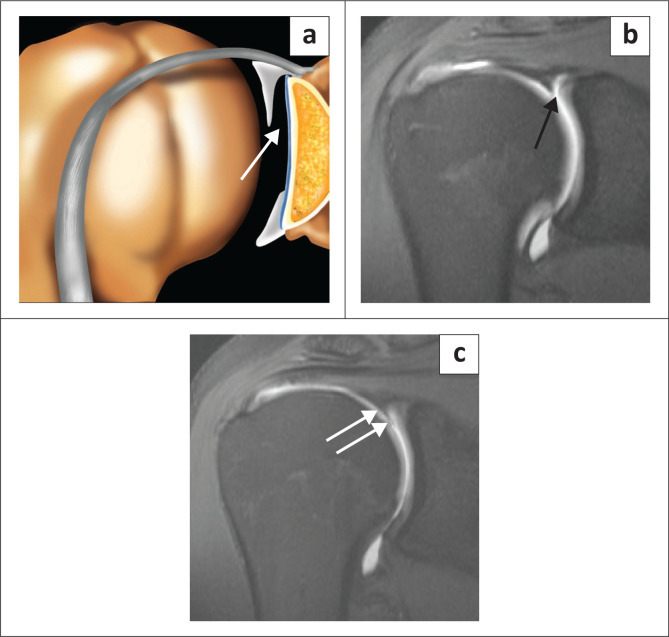
Type 3 biceps labral complex with a larger recess (arrow in b) and a meniscoid (double arrows in c) appearance to the labrum as seen on two consecutive coronal oblique images on this FS T1W MR arthrogram study. Meniscoid appearance of the labrum is clearly displayed in the colour illustration in (a).

The superior labrum is contiguous with the biceps tendon and is known as the biceps labral complex (BLC). There are three types of attachment of the BLC to the glenoid^18,19,20^ and these are best assessed on coronal images. In the type 1 BLC, the BLC is firmly adherent to the superior glenoid rim with no recess present (Figure 5). In the type 2 BLC, there is a small recess between the labrum and cartilage (Figure 6) and in the type 3 BLC, there is a meniscoid labrum with a large recess (Figure 7).

**FIGURE 8:**
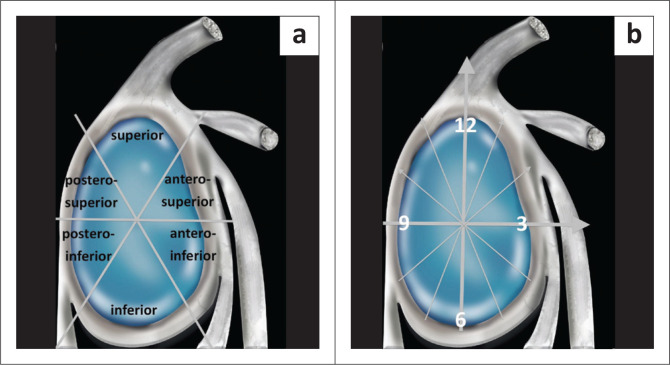
Nomenclature for localisation of a labral tear. (a) Quadrant description. (b) Clock zone description.

The glenoid labrum is divided into six quadrants (Figure 8a) or clock zones (Figure 8b) to describe the location of the lesions.^3,13,14,18^ In the clock zone description, the labrum is likened to the face of a clock with the superior pole referred to as the 12 o’clock position and the inferior pole as the 6 o’clock position. By convention, 3 o’clock refers to the anterior portion and 9 o’clock to the posterior portion in both shoulders.

## Anatomic variants of the superior and anterosuperior labrum

There are three well-recognised anatomic variants located in the superior and anterosuperior quadrants, namely the sublabral recess, the sublabral foramen and the Buford complex. The bicipital labral sulcus or pseudo-SLAP lesion and high attachment of the anterior band of the IGHL represent additional important anatomic variants (Box 1).

BOX 1 Anatomic variants of the superior and anterosuperior labrum.

**Table:** 

Important variants of the Glenoid Labrum
Sublabral recess or sulcus
Sublabral foramen or hole
Buford complex
Biciptal labral sulcus or pseudo SLAP lesion
High attachment of anterior band of inferior glenohumeral ligament

SLAP, superior labrum anterior to posterior.

### Sublabral recess or sulcus

**FIGURE 9:**
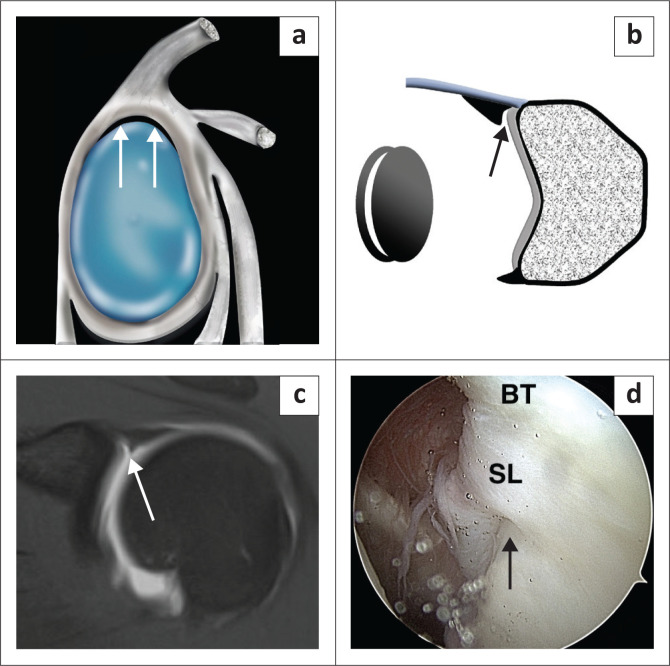
Sublabral recess or sulcus. (a) Sagittal colour illustration of a sublabral recess. (b) Coronal illustration depicting a sublabral recess (arrow) and the single ‘Oreo cookie’ sign. (c) FS T1W MR arthrogram coronal oblique view depicting a sublabral recess (arrow) in this 20-year-old male with a posterior labral tear (not shown). (d) Arthroscopy image confirming a sublabral recess (arrow) (BT, biceps tendon; SL, superior labrum).

The sublabral recess or sulcus is the most common anatomic variant of the superior labrum (Figure 9) and has been identified in 73% of shoulders in cadaver studies.^21^ This variant is located in the superior quadrant at the 11 to 1 o’clock positions and represents the type 2 or 3 BLC attachment described earlier. The recess may co-exist with a sublabral foramen or hole.^19^ This variant is best assessed in the coronal oblique plane (Figure 9c) and should measure no more than 2 mm on conventional MR (2.5 mm on direct MR arthrogram) in its mediolateral dimension in this plane^6,7,9,13,14^ although Stoller indicates that a normal recess can measure up to 5 mm.^18^ The appearance of this recess has been likened to a single ‘Oreo cookie’ (Figure 9b).^3^

The classic cadaver study of De Palma et al.^22^ made two pertinent observations: (1) The absence of a sublabral recess in foetuses and infants and (2) the increasing frequency of a sublabral recess with advancing age. A recess was identified in 17%, 50% and 95% of specimens derived from persons in the second decade, persons older than 20 years and persons in the seventh and eighth decades, respectively. In a more recent study by Fealey et al.,^23^ a recess was observed in fetuses over 22 weeks gestational age. There is no clarity at which age normal labral separation occurs but there is agreement on the presence of an area of loose anterosuperior labral attachment. This area may progress to physiological separation (sublabral recess) and may be converted to pathological detachment (SLAP lesion) with excessive stress.^3,14^

The importance of this variant is that it can be mistaken for a SLAP II lesion and, in some cases, a recess cannot be differentiated from a SLAP tear. There are certain important features that help to differentiate a recess from a tear:

- Location: a recess typically extends only to the most posterior insertion point of the biceps tendon attachment to the labrum and glenoid. Jin et al.^24^ have however demonstrated that a recess may extend posterior to the biceps tendon attachment.
- Size: a recess has a width up to 2.5 mm whereas a tear has a width of more than 2.5 mm on direct MR arthrogram (or 2 mm on conventional MRI).^6,7,9,13,14^
- Contour: a sulcus should demonstrate smooth margins. Contour irregularity should be considered suspicious for SLAP tear.^25^
- Orientation: the direction of increased signal intensity or fluid should extend medially, paralleling the underlying glenoid cartilage; any extension laterally into the substance of the labrum should be considered suspicious for a tear.^25^
- Superior labrum: a recess maintains a normal triangular superior labrum with a well-defined edge and no abnormal signal within its substance. A SLAP tear may have an irregular superior labrum with abnormal intrasubstance signal.

### Sublabral foramen or hole

**FIGURE 10:**
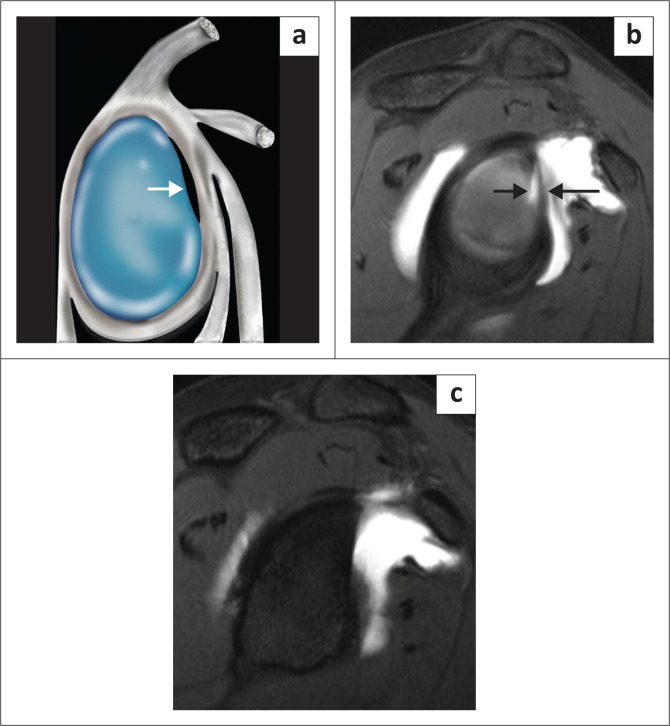
Sublabral foramen or hole: (a) Colour illustration of a sublabral foramen. (b) Short black arrow denotes the sublabral foramen and long black arrow the detached labrum on this FS T1W MR arthrogram sagittal oblique image. (c) Note the pear-shaped appearance of the glenoid in the same patient on this FS T1W MR arthrogram sagittal oblique image.

**FIGURE 11:**
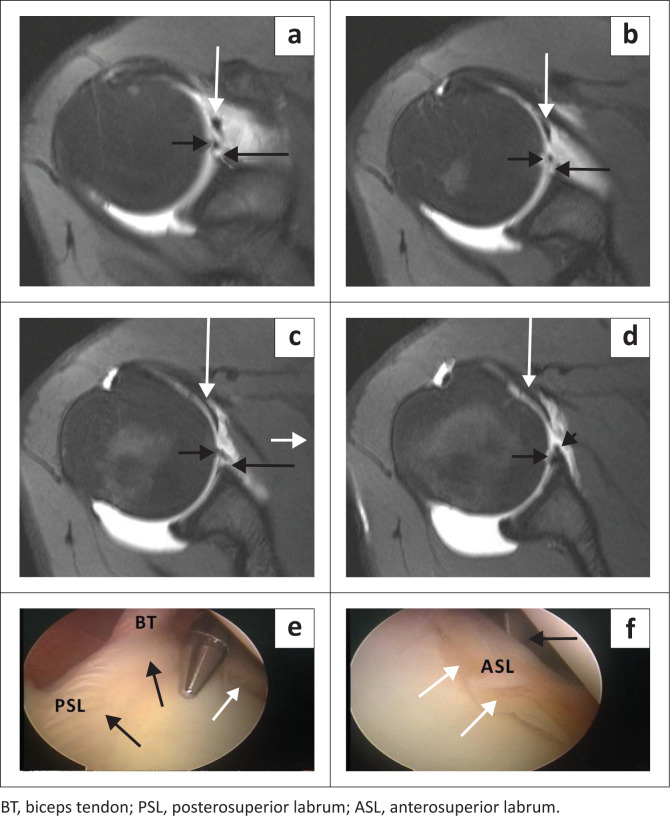
Sublabral foramen or hole: (a–d) Four consecutive axial images from superior to inferior (FS T1W MR arthrogram) demonstrating a sublabral foramen (long black arrow) and adjacent detached anterosuperior labrum (short black arrow). Note the point of reattachment of the labrum (black arrowhead in d). The white arrow represents the middle glenohumeral ligament; (e–f) Arthroscopic (posterior portal) confirmation of a sublabral foramen (white arrow) in this 16-year-old water polo player. Note the firmly attached superior and posterosuperior labrum (PSL) in (e) (black arrows). Arthroscopic probe (black arrow in f) displaces the detached anterosuperior labrum (ASL).

**FIGURE 12:**
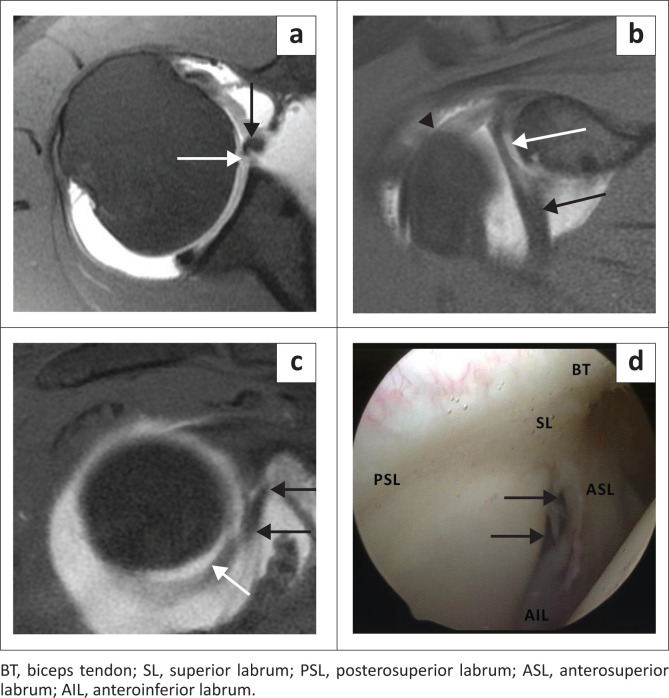
Sublabral foramen or hole: (a) FS T1W MR arthrogram axial image showing a sublabral foramen (white arrow) and non-pathological detachment of the anterior labrum (black arrow). (b) FS T1W MR arthrogram coronal oblique image showing a sublabral foramen (white arrow). Anteroinferior labrum represented by black arrow and biceps tendon by black arrowhead. (c) FS T1W MR arthrogram sagittal oblique image demonstrates firm attachment of the anteroinferior labrum (white arrow) and loose attachment of the anterosuperior labrum (black arrows). (d) Arthroscopic (posterior portal) image confirming a sublabral foramen (black arrows) in this patient with a supraspinatus tear (not shown).

The sublabral foramen or hole is seen in 11% – 17% of individuals and consists of the lack of attachment of the labrum to the glenoid rim in the anterosuperior quadrant at the 1 to 3 o’clock positions (Figure 10).^18^ This variant is often seen with a pear-shaped glenoid fossa (Figure 10c)^19^ It may co-exist with the sublabral recess^19^ and may be associated with a cord-like middle glenohumeral ligament (MGHL). It is best assessed in the axial imaging plane (Figure 11 and Figure 12). The sublabral foramen may be mistaken for an anterior labral tear. Features that help to differentiate it from a tear include:

- Location: typically, at the 1–3 o’clock position. A study by Tuite et al.,^26^ however, indicated that the labral detachment may extend beyond the mid-glenoid notch or 3 o’clock position.
- Contour: the foramen or detachment should have smooth margins.^20^
- Displacement: the labral displacement should be < 2 mm.^21^
- Absence of injury to the adjacent capsuloligamentous structures.^20^

### Buford complex

**FIGURE 13:**
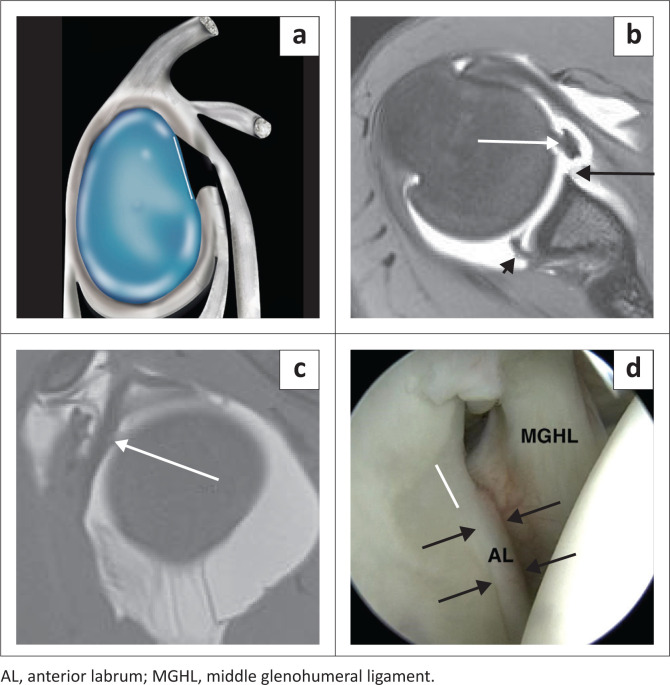
Buford complex: (a) Colour illustration of the Buford complex depicting absent labral tissue (white line). (b) FS T1W MR arthrogram axial view depicting the absent labrum anteriorly (black arrow) and the cord-like middle glenohumeral ligament (MGHL) (white arrow). Posterosuperior labral tear depicted by a black arrowhead. (c) FS T1W MR arthrogram sagittal oblique view demonstrating a cord-like MGHL (white arrow). (d) Arthroscopy image confirming the Buford complex in a 20-year-old male with a posterosuperior labral tear. The white line depicts absent labral tissue in the anterosuperior quadrant.

The Buford complex is seen in 1.5% of individuals and consists of three defining elements: an absent anterosuperior labrum, a cord like MGHL and a MGHL that attaches directly to the superior labrum anterior to the biceps (at the base of the biceps anchor).^18^ This variant is best assessed by identifying the cord like MGHL on consecutive axial images and cross-referencing on sagittal images (Figure 13).^20^ A Buford complex should be suspected if the contiguous anteroinferior and superior labrum appear normal.^27^ It is important to recognise this uncommon variant as the absent anterosuperior labrum and cord like MGHL may be mistaken for an anterior labral tear and a displaced long head of biceps tendon, respectively.^20^ Failure to recognise the Buford complex may also result in inappropriate surgical attachment of the MGHL to the anterior glenoid with resultant limitation of shoulder elevation and external rotation.^18^

### Bicipital labral sulcus or pseudo-superior labrum anterior to posterior lesion

**FIGURE 14:**
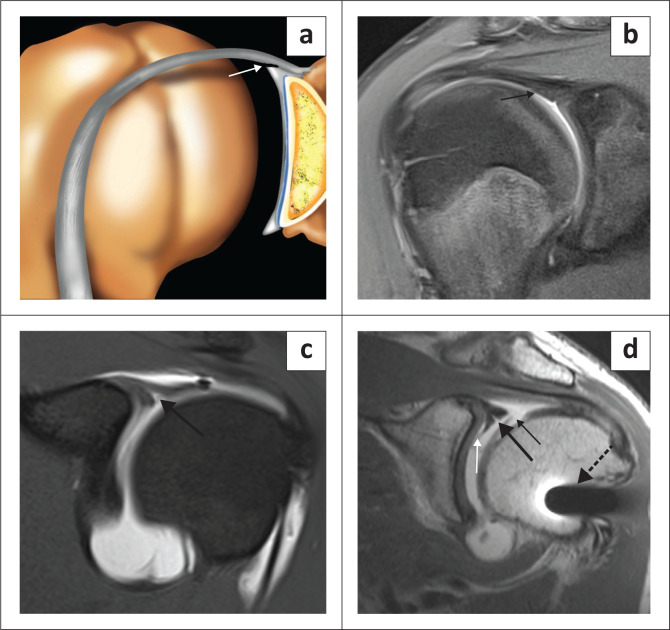
Bicipital labral sulcus or pseudo-superior labrum anterior to posterior tear: (a) Colour illustration of the bicipital labral sulcus depicted by a white arrow. (b) Fat-sat proton density (FS PD) coronal image in a 15-year-old water polo player depicting a very small sulcus (black arrow). (c) FS T1 arthrogram coronal image demonstrating a deep sulcus (black arrow) that may be misinterpreted as a tear. (d) PD arthrogram coronal image showing a small bicipital sulcus (thick black arrow) and a sublabral recess (thick white arrow) with a meniscoid appearance to the labrum (type 3 biceps labral complex attachment). The thin black arrow denotes a humeral chondral fracture and the dashed arrow relates to an internal fixation screw.

There is a normal lateral oblique sulcus or cleft of fluid signal intensity between the biceps tendon and the superior labrum anterior to the biceps labral junction (Figure 14a).^18,20,28^ The fluid or contrast-filled sulcus can be of variable depth and a deep sulcus can resemble a SLAP tear, hence the term pseudo-SLAP tear (Figure 14b–d).^28^ Helpful differentiating features from a tear include the smooth margins of the sulcus with no abnormal signal traversing the labrum whereas a tear will typically demonstrate irregular margins and abnormal signal within the labrum.

### High attachment of the anterior band of the inferior glenohumeral ligament

**FIGURE 15:**
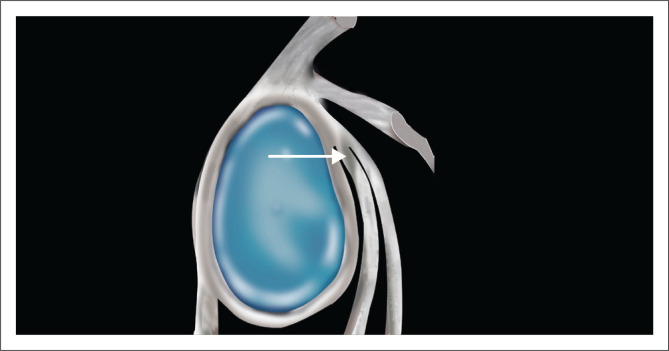
High insertion of the anterior band of the inferior glenohumeral ligament (IGHL). White arrow denotes the high attachment of the IGHL above the equator in contradistinction to the more common attachment site below the equator as seen in Figure 1.

A less well-recognised variant is that of the high attachment of the anterior band of the IGHL (Figure 15). This entity is well-described by Stoller.^18^ The prominent anterior band is identified above the equator and can exist with the following variations: (1) with a small anterosuperior labrum, (2) with an absent anterosuperior labrum and (3) with a cord-like MGHL associated with an absent or small anterosuperior labrum.

**FIGURE 16:**
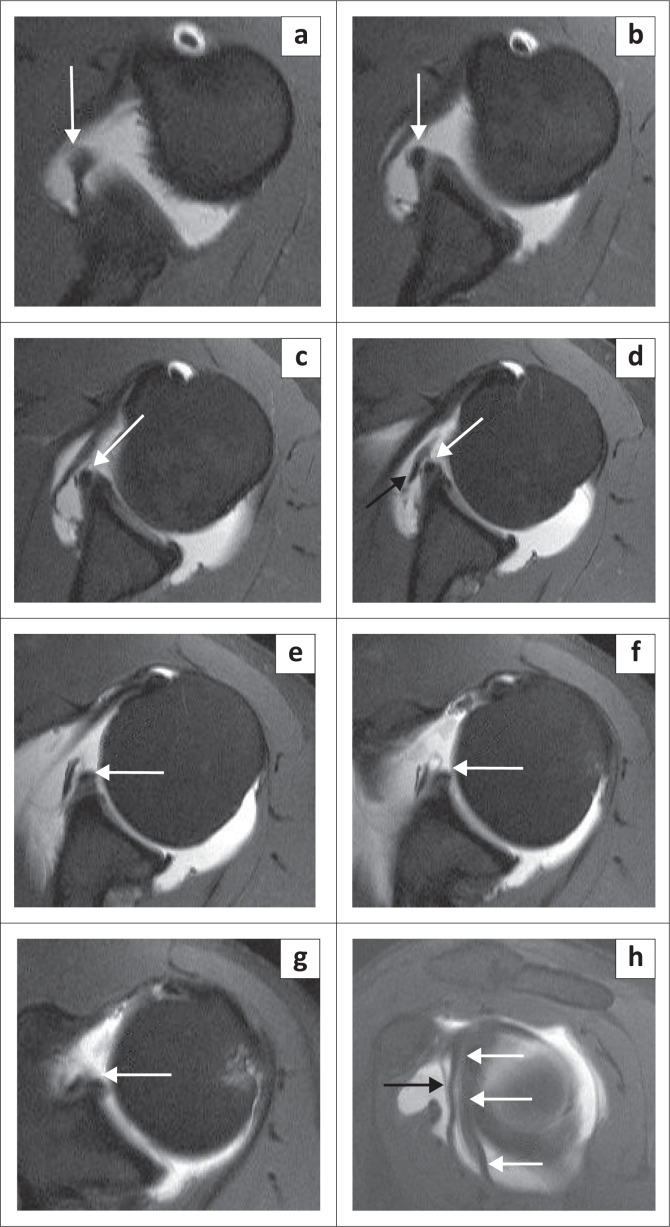
High insertion of the anterior band of the inferior glenohumeral ligament (IGHL): (a–g) The course of the anterior band of the IGHL (white arrow) is clearly shown on consecutive axial images (FS T1W MR arthrogram) from inferior to superior. (h) Sagittal oblique image (FS T1W MR arthrogram) demonstrating the high attachment of IGHL (white arrow). A black arrow denotes the middle glenohumeral ligament.

At arthroscopy, this high attachment of the IGHL may appear to create a sublabral foramen if the anterosuperior labrum is absent. Stoller is of the opinion that in many cases, the diagnosis of a sublabral foramen in fact represents a high attachment of the anterior band of the IGHL with an absent anterosuperior labrum.^18^ This variant can, as with the sublabral foramen, be misinterpreted as representing a displaced anterior labral tear. It is best diagnosed on axial images by identifying the anterior band of the IGHL in the inferior part of the joint and following its course superiorly to its attachment site above the equator as depicted in Figure 16.

Several studies have demonstrated that these variants may predispose to pathological conditions.^7^ The presence of a sublabral foramen and a non-cordlike MGHL had a higher prevalence of type II SLAP lesions.^29^ The importance of these variants is that they can be mistaken for SLAP tears, particularly the sublabral recess variant, with resultant unnecessary surgery. In some cases, a repair of, for example, a Buford complex may result in an adverse outcome.^18^

## Conclusion

The glenoid labrum is optimally evaluated by direct MRI arthrography. Knowledge of the labral anatomy including the anatomic variants is essential in the evaluation of a patient for a possible SLAP lesion as these variants can be mistaken for SLAP tears.
